# UNIVERSAL FAMILY CARE: DECISION POINTS FOR STATES IN IMPROVING THE CARE INFRASTRUCTURE THROUGH SOCIAL INSURANCE

**DOI:** 10.1093/geroni/igz038.2122

**Published:** 2019-11-08

**Authors:** Benjamin Veghte, Alexandra L Bradley, Marc Cohen, Heidi Hartmann, Benjamin Veghte

**Affiliations:** 1 National Academy of Social Insurance, Washington, District of Columbia, United States; 2 University of Massachusetts, Boston, Boston, Massachusetts, United States; 3 Institute for Women’s Policy Research, Washington, District of Columbia, United States; 4 Caring Across Generations, Washington, District of Columbia, United States

## Abstract

This report presents policy options for a new social insurance program – akin to Social Security or Medicare – that would provide an integrated approach to protecting against the risk of needing to provide or receive care across the lifespan: Universal Family Care. The program would allow individuals and families to access caregiving supports at various points throughout the life course, from the arrival of a child to end-of-life care for a family member or oneself. The program covers three specific caregiving needs: long-term services and supports, paid leave, and early child care and education. This report identifies key design questions for states to consider in crafting a UFC program, outlines a range of vetted approaches, and describes the building blocks and tradeoffs involved. This analysis was developed during a year of deliberations by a Study Panel of 30 experts in care policies from a variety of perspectives.

